# Extracts, Anthocyanins and Procyanidins from *Aronia melanocarpa* as Radical Scavengers and Enzyme Inhibitors

**DOI:** 10.3390/nu5030663

**Published:** 2013-03-04

**Authors:** Marie Bräunlich, Rune Slimestad, Helle Wangensteen, Cato Brede, Karl E. Malterud, Hilde Barsett

**Affiliations:** 1 School of Pharmacy, Department of Pharmaceutical Chemistry, University of Oslo, P.O. Box 1068, Blindern, N-0316 Oslo, Norway; E-Mails: helle.wangensteen@farmasi.uio.no (H.W.); k.e.malterud@farmasi.uio.no (K.E.M.); hilde.barsett@farmasi.uio.no (H.B.); 2 PlantChem, Særheim Research Center, N-4353 Klepp station, Norway; E-Mail: rune@plantchem.com; 3 Department of Medical Biochemistry, Stavanger University Hospital, N-4068 Stavanger, Norway; E-Mail: cabr@sir.no

**Keywords:** *Aronia melanocarpa*, anthocyanins, procyanidins, DPPH, 15-lipoxygenase, xanthine oxidase, α-glucosidase

## Abstract

Extracts, subfractions, isolated anthocyanins and isolated procyanidins B2, B5 and C1 from the berries and bark of *Aronia melanocarpa* were investigated for their antioxidant and enzyme inhibitory activities. Four different bioassays were used, namely scavenging of the diphenylpicrylhydrazyl (DPPH) radical, inhibition of 15-lipoxygenase (15-LO), inhibition of xanthine oxidase (XO) and inhibition of α-glucosidase. Among the anthocyanins, cyanidin 3-arabinoside possessed the strongest and cyanidin 3-xyloside the weakest radical scavenging and enzyme inhibitory activity. These effects seem to be influenced by the sugar units linked to the anthocyanidin. Subfractions enriched in procyanidins were found to be potent α-glucosidase inhibitors; they possessed high radical scavenging properties, strong inhibitory activity towards 15-LO and moderate inhibitory activity towards XO. Trimeric procyanidin C1 showed higher activity in the biological assays compared to the dimeric procyanidins B2 and B5. This study suggests that different polyphenolic compounds of *A. melanocarpa* can have beneficial effects in reducing blood glucose levels due to inhibition of α-glucosidase and may have a potential to alleviate oxidative stress.

## 1. Introduction

Aronia, *Aronia melanocarpa* (Michx.) Elliott, syn. *Photinia melanocarpa* (Michx.) K. R. Robertson and J. B. Phipps, sometimes called black chokeberry, belongs to the Rosaceae family and is cultivated as a decorative shrub, as a source of berries for juices, wines and jams and as a rich source of natural food colorants [1,2]. In recent years, black chokeberries have gained popularity due to their high content of polyphenols with antioxidant activity [3]. In fact, they possess the highest antioxidant activity among berries and other fruits investigated so far as measured with the oxygen radical scavenging capacity (ORAC) assay [2,4]. The Aronia berries contain high levels of flavonoids, mostly anthocyanins and proanthocyanidins. The formation of free radicals is strongly associated with lipid peroxidation and has also been implicated in the development of a variety of diseases, including cellular aging, mutagenesis, inflammation, carcinogenesis, coronary heart disease and diabetes [5]. Accumulated evidence has suggested that diabetic patients are under oxidative stress, with an imbalance between the free radical generating and radical scavenging capacities [6,7,8,9,10,11]. 15-Lipoxygenase (15-LO) and xanthine oxidase (XO) are peroxidative and prooxidative enzymes, respectively, and sources of reactive oxygen species (ROS) in vascular cells [12,13]. An overproduction of ROS may be involved in endothelial dysfunction. Thus, substances that inhibit the production of ROS could have a positive effect on cardiovascular function. It has been suggested that *A. melanocarpa* fruit juice and its anthocyanins might be useful in the prevention and control of diabetes mellitus type II and diabetes associated complications [14,15]. α-Glucosidase, which is a membrane-bound enzyme located at the epithelium of the small intestine, plays a vital role in digestion of carbohydrates, as it catalyzes the cleavage of glucose from disaccharides and oligosaccharides. It might be possible to prevent the onset of diabetes by controlling postprandial hyperglycemia through the inhibition of α-glucosidase and α-amylase activities, resulting in a delay of carbohydrate digestion to absorbable monosaccharide [16]. Studies have revealed that anthocyanins potentially inhibit intestinal α-glucosidase, and Adisakwattana *et al*. [16] reported that cyanidin 3-rutinoside retards absorption of carbohydrates by that mechanism of action. To our knowledge, no systematic investigation on chokeberry anthocyanins and procyanidins as radical scavengers, lipoxygenase inhibitors, xanthine oxidase inhibitors and α-glucosidase inhibitors has been reported previously.

The present paper reports *in vitro* antioxidant activity of extracts, subfractions, anthocyanins and procyanidins isolated from *A. melanocarpa* measured by scavenging of 1,1-diphenyl-2-picrylhydrazyl (DPPH) radical and inhibition of the enzymes 15-LO and XO. Furthermore, the polyphenol-rich extracts were tested for their ability to inhibit α-glucosidase.

## 2. Experimental Section

### 2.1. Plant Material

Aronia berries, *A. melanocarpa* (Michx.) Elliott var. Moscow, were harvested at Særheim, Klepp, Norway (58°47′N, 5°41′E) in August 2010. The berries were kept at −20 °C until extraction. A voucher specimen (MB201201) is deposited in the Pharmacognosy section, School of Pharmacy, University of Oslo, Norway. Bark, as a source of procyanidins, was sampled from the same plants. Branches with a diameter of 1–2 cm were chosen, and the bark was carefully removed. In total 1500 g fresh weight (FW) was sampled, giving 732 g dry weight (DW) upon lyophilization. The plant material was cut in pieces and kept at −20 °C until extraction.

### 2.2. Chemicals

Diphenylpicrylhydrazyl (DPPH) radical, linoleic acid, 15-LO from soybeans, hypoxanthine, XO from bovine milk, α-glucosidase from baker’s yeast, 4-nitrophenyl α-D-glucopyranoside (PNP-G), quercetin, acarbose, sodium potassium phosphate, benzyl mercaptan, trifluoroacetic acid (TFA), dimethyl sulfoxide (DMSO), ethyl acetate (EtOAc), dichloromethane (DCM), acetone, ethanol (EtOH) and methanol (MeOH) were purchased from Sigma-Aldrich (St. Louis, MO, USA). Procyanidin B2 and B5 reference compounds were obtained from Plant Polyphenols LLC (Boyce, LA, USA). All other reagents were of the highest purity available.

### 2.3. Extraction and Fractionation

Aronia berries (5.5 kg FW) were freeze-dried, pulverized and extracted with DCM (5 L) followed by EtOH (4.5 L) in a Soxhlet apparatus. The plant residue was further extracted by stirring with 3 × 3 L 50% EtOH at 70 °C for 2 h. All extracts were concentrated *in vacuo*, yielding 26 g, 250 g and 80 g, respectively. The 50% EtOH extract (2.5 g) was fractionated on an Amberlite XAD-7HP (3 × 30 cm; Sigma-Aldrich, St. Louis, MO, USA) column by elution with 400 mL water followed by 300 mL MeOH, 200 mL 0.1% TFA in MeOH and 200 mL acetone-water (70:30). The procedure was repeated until a total amount of 10 g had been applied on the column. The MeOH fraction (Amb-MeOH) was concentrated to dryness *in vacuo*, and a yield of 2 g was obtained. Of this, 920 mg was dissolved and further fractionated over Sephadex LH-20 (3 × 30 cm; GE Healthcare, Uppsala, Sweden) with MeOH-water (from 20:80 to 100:0) as eluent. This gave three subfractions, namely Seph I (418 mg; eluted with 20% MeOH), Seph II (44 mg; 40% MeOH) and Seph III (278 mg; 100% MeOH).

### 2.4. Isolation of Anthocyanins

In parallel to the work described in section 2.3., Aronia berries (2 kg FW) were extracted by maceration with 2 × 6 L MeOH (0.5% TFA v/v) for 24 h at ambient temperature and most of the solvent evaporated *in vacuo*. The concentrated water-enriched extract (0.5 L) was partitioned against EtOAc (4 × 0.5 L). The organic phase was discarded, whereas the water-phase was concentrated and purified on a bed of Amberlite XAD-7HP (5 × 50 cm column) by use of water until the eluate had pH 6 followed by elution with 1 L MeOH (0.5% TFA). The anthocyanin-enriched extract was then eluted through a Sephadex LH-20 column (5 × 100 cm) by use of a step gradient of 15% (3 L) and 30% (4 L) MeOH (0.1% TFA v/v). Fractions of 200 mL were collected and analyzed by HPLC. Purity acceptance was defined as the analyte peak area representing >96% of total area as detected at 520 and 280 nm. Overlapping fractions were combined and reapplied to the same column, and five column runs gave approximately 200 mg cyanidin 3-galactoside (**1**), 200 mg cyanidin 3-arabinoside (**3**) and 20 mg cyanidin 3-xyloside (**4**). Only minute amounts of pure cyanidin 3-glucoside (**2**) were obtained. 

### 2.5. Isolation of Procyanidins

Instead of using berries as a source of oligomeric procyanidins, bark was chosen, as this is a richer source and as this gave a simpler polyphenolic composition (e.g., no anthocyanins). A bark sample of 466 g DW was extracted with 2 × 10 L 70% acetone at room temperature for 24 h. After concentration *in vacuo*, the 0.5 L extract was defatted with 2 × 0.5 L DCM. The organic phase, which contained 6.4 g dry matter, was discarded. The sample was further partitioned against 3 × 0.5 L EtOAc, and the organic phase was concentrated *in vacuo*, followed by lyophilization. The EtOAc part yielded 7.8 g DW. Of this, 2 g was applied to a 5 × 100 cm column of Sephadex LH-20, and separation was achieved by use of a step gradient of 50% (2 L), 80% (3 L) and 100% (10 L) MeOH. Fractions of 1 L were collected and analyzed by thin layer chromatography (TLC) and HPLC. About 200 mg procyanidin B2 (**5**), 20 mg B5 (**6**) and 50 mg of C1 (**7**) were obtained. Purity acceptance was defined as the analyte peak area comprising >96% of total chromatographic area at 280 nm and at fluorescence detection (see section 2.6.).

### 2.6. Analysis

#### 2.6.1. TLC

TLC analysis of procyanidins was performed with silica gel on polyethylene terephthalate (PET)-foils (Sigma-Aldrich, St. Louis, MO, USA) in the mobile phase system EtOAc-HCOOH-HOAc-H_2_O (75:2:3:20 v/v), upper layer. Mobile-phase distance was 10 cm. Spraying with vanillic acid-HCl-MeOH (1:4:100, w/v/v), followed by heating with a hair dryer, was used for spot detection.

#### 2.6.2. HPLC

An Agilent 1100-system, Agilent Technologies (Santa Clara, CA, USA), was used for the purity check of the individual anthocyanins, and they were identified by comparison with in-house reference compounds [17]. Separation took place over an Eclipse XDB-C8 (4.6 × 150 mm, 5 µm) column (Agilent Technologies, Santa Clara, CA, USA) by use of a binary solvent system consisting of (A) HCOOH-H_2_O (1:9 v/v) and (B) HCOOH-MeOH-H_2_O (10:50:40, v/v). The gradient (B in A) was isocratic, with 0% B for 2 min, linear from 0% to 70% in 18 min, from 70% to 100% for the next 2 min, from 100% to 0% in 2 min, followed by recondition of the column for 2 min. All HPLC samples were filtered through a 13 mm syringe filter (Nylon 0.45 µm, VWR International, Radnor, PA, USA) prior to injection. The flow rate was 0.8 mL/min, 10 µL samples were injected on the column and separation took place at 30 °C. Chromatograms were obtained at 280 and 520 nm.

HPLC analyses of procyanidins were performed on the same instrument and column with the solvent system (A) 0.05% TFA and (B) 0.05% TFA in MeCN. The gradient (B in A) was 5% (10 min), from 5% to 15% (10 min), from 15% to 20% (10 min), from 20% to 100% (6 min), from 100% to 5% (4 min) and finally recondition of the column for 2 min. The flow rate was 0.5 mL/min, column temperature 30 °C, and aliquots of 10 μL were injected. Fluorescence detection was achieved with excitation at 276 nm and emission at 316 nm (HP 1046A detector, Hewlett-Packard, Palo Alto, CA, USA), whereas UV-absorbance was detected at 280 nm. 

#### 2.6.3. UV Measurements

For UV measurements used in bioassay, a Biochrom Libra S32 PC (Biochrom Ltd, Cambridge, UK) was employed. 

#### 2.6.4. Thiolysis

About 10 mg crude extract was dissolved in 5% benzyl mercaptan in MeOH containing 1.1% HCl (v/v) and kept at 50 °C for 30 min. Procyanidin B1 and B2 were used as standards, with terminal units catechin and epicatechin, respectively. The products following the thiolysis reactions were analyzed by the method described for procyanidins on HPLC.

#### 2.6.5. Mass Spectrometry

Crude extracts of berries (MeOH-extract) and bark (70% acetone), together with the isolated procyanidins, were further characterized by liquid chromatography coupled with mass spectrometry (LC-MS) using a nanoAcquity ultra performance liquid chromatography (UPLC) (Waters, Milford, MA, USA) coupled with a quadrupole time-of-flight (QTOF) micro hybrid mass spectrometer (Waters) equipped with nanoLockspray mass calibration. Reversed phase separations were achieved on a 600 mm long and 0.05 mm ID in-house prepared porous polymer monolithic column. The mobile phase consisted of (A) 0.1% formic acid and 0.05% ammonium hydroxide in water mixed with (B) 0.1% formic acid in acetonitrile. Injection of 2 µL sample diluted in water was done with a high flow rate (3 µL/min) for 5 min on a short trap column (50 mm) of a similar type as the analytical column, using a mobile phase composition of 1% B. A linear gradient from 0 to 10 min was performed by varying B from 2% to 10% at an elevated flow rate of 400 nL/min, in order to compensate for the large dwell volume. Then, the flow rate was reduced to 200 nL/min and the compounds eluted with a gradient reaching 30% of B at 30 min and 80% of B at 40 min. Electrospray ionization in the positive mode (ESI+) was used with a capillary voltage at 3 kV and a cone voltage at 35 V. Mass spectra were collected in the *m/z* range of 200–2000 with 1 s scantime. Instrument control, data acquisition and data processing were done by using MassLynx 4.1 software (Waters, Milford, MA, USA).

#### 2.6.6. NMR

^1^H and ^13^C nuclear magnetic resonance (NMR) spectroscopy of extract and fractions were conducted on Varian Gemini 200 (Varian, Palo Alto, CA, USA), Bruker DPX 300 or Bruker AVII 400 (Bruker, Rheinstetten, Germany) instruments and performed in CDCl_3_ or CD_3_OD with tetramethylsilane (TMS) as an internal standard. 

### 2.7. DPPH Radical Scavenging

Scavenging activity towards the DPPH radical was carried out as previously described [18]. Briefly, to 2.95 mL of a methanolic solution of DPPH (A_517_ 1.0), 50 µL of the test compound (dissolved in DMSO or MeOH) was added. The mixture was stirred, and the decrease in UV absorbance at 517 nm was measured over a period of 5 min. Percent radical scavenging was calculated as 100 × (A_start_ − A_end_)/(A_start_), where A_start_ is the absorbance before addition of the test compound and A_end_ is the absorbance value after 5 min of reaction time. Values were corrected for absorbance of the test substances. Quercetin was used as a positive control.

### 2.8. Inhibition of 15-Lipoxygenase (15-LO)

Soybean lipoxygenase was used to measure inhibition of 15-LO. To a solution of linoleic acid (134 µM) in borate buffer (0.2 M, pH 9.00, 2.90 mL) was added 50 µL of test substance dissolved in DMSO or MeOH or (for blanks) DMSO or MeOH alone. A solution of 15-LO in 50 µL borate buffer (10,000 U/mL) was added, and the increase in absorbance at 234 nm from 30 to 90 s after addition was measured. Percent enzyme inhibition was calculated as 100 × [(∆A_1_ − ∆A_2_)/∆A_1_], where ∆A_1_ and ∆A_2_ are values for increase in A_234_ for sample without test substance and with test substance, respectively [18]. Quercetin was used as a positive control.

### 2.9. Inhibition of Xanthine Oxidase (XO)

The XO inhibitory activity with hypoxanthine as the substrate was measured spectrophotometrically based on the procedure of Noro *et al.* [19], with some modifications. The assay mixture consisting of 50 µL of test compound (dissolved in DMSO or MeOH) or (for blanks) DMSO or MeOH alone, 1.85 mL of 50 mM sodium-potassium phosphate buffer (pH = 7.5) and 100 µL of enzyme solution (1.8 U/mL in 50 mM sodium-potassium phosphate buffer, pH = 7.5) was prepared immediately before use. Substrate solution (1.0 mL, 20 µg/mL hypoxanthine in distilled water) was added. The mixture was stirred, and the increase in absorbance at 290 nm was measured over a period of 5 min. Percent enzyme inhibition was calculated as 100 × [(∆A_1_ − ∆A_2_)/∆A_1_], where ∆A_1_ and ∆A_2_ are values for the increase in A_290_ for the sample without test substance and with test substance, respectively. Quercetin was used as a positive control.

### 2.10. α-Glucosidase Inhibitory Activity

The α-glucosidase inhibitory activity was determined by a slight modification of the procedure reported by Matsui *et al.* [20]. The enzyme solution was set at 800 mU/mL of α-glucosidase in a 50 mM phosphate buffer (pH = 7.0) containing 100 mM sodium chloride. For each assay, 20 µL of the test solution in DMSO or MeOH and 80 µL of the enzyme solution were preincubated at 37 °C for 5 min. The reaction was started by adding 1.9 mL of substrate solution (0.7 mM PNP-G in the buffer), and the solution was then incubated at 37 °C for 15 min. After the reaction had been stopped by adding 2.0 mL of a 0.5 M Tris solution, the absorbance of PNP released from PNP-G at 400 nm was measured. Percent enzyme inhibition was calculated as 100 × (A_B_ − A_S_)/(A_B_), where A_B_ and A_S_ represent the absorbance of the blank and sample, respectively. Acarbose was used as a positive control.

### 2.11. Statistics

Samples for DPPH, 15-LO, XO and α-glucosidase assays were analyzed in triplicate and results are given as averages ± SD. Student’s *t* test was used for statistical evaluation and *p* < 0.05 was considered statistically significant. 

## 3. Results and Discussion

### 3.1. Extraction and Chemical Characterization

**Figure 1:**
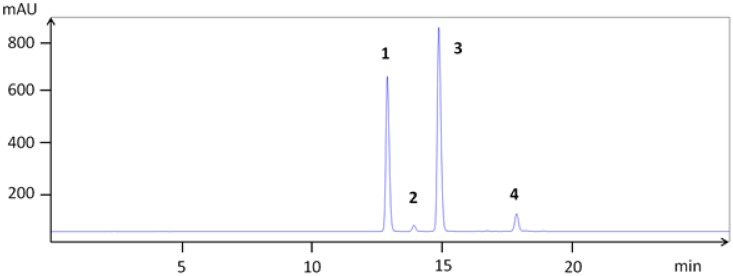
High-performance liquid chromatography (HPLC) chromatogram of the isolated anthocyanins: cyanidin 3-galactoside (**1**), cyanidin 3-glucoside (**2**), cyanidin 3-arabinoside (**3**) and cyanidin 3-xyloside (**4**).

**Figure 2:**
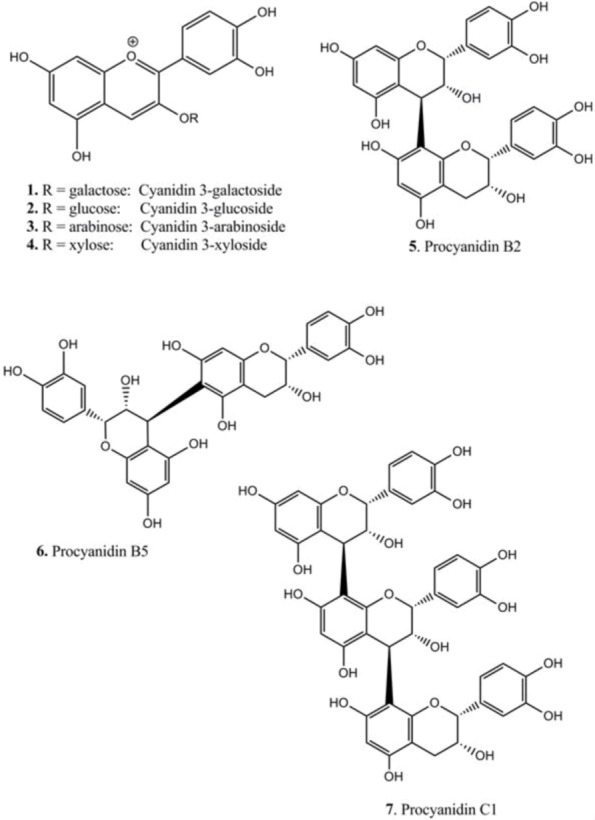
Chemical structures of compounds isolated from berries and bark of Aronia.

**Figure 3:**
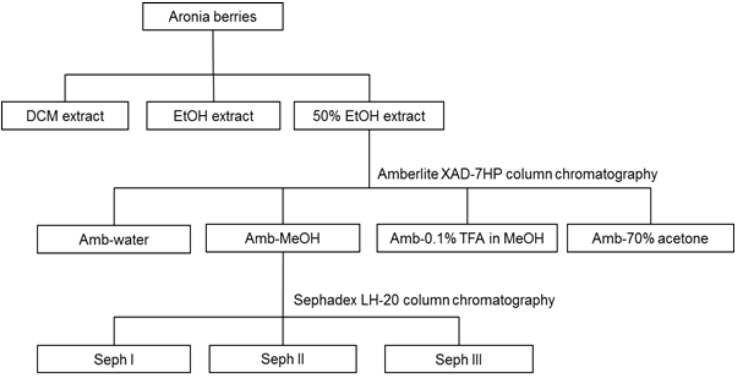
Procedure for extraction and fractionation from berries of Aronia. Anthocyanins (section 2.4.) and procyanidins (section 2.5.) were extracted by different procedures.

In chokeberry fruits, anthocyanins constitute the second largest group of phenolic compounds [1]. The anthocyanins in *A. melanocarpa* are mainly a mixture of four cyanidin glycosides: 3-galactoside, 3-glucoside, 3-arabinoside and 3-xyloside, of which cyanidin 3-galactoside is the main one (Figure 1) [17]. In order to isolate the major anthocyanins from Aronia berries, extraction with MeOH containing TFA was performed, since direct alcoholic extractions provide very poor yield, not keeping anthocyanins in the stable flavylium cationic form. The anthocyanins (**1**–**4**) were isolated as pure compounds, and their structures are shown in Figure 2. Their chromatographic and spectral characteristics were in agreement with previous observations [17]. Aronia berries were also extracted as shown in Figure 3.

^1^H NMR and ^13^C NMR analysis revealed that proanthocyanidins were present in the 50% EtOH extract (70 °C). Subfractions of the extract were further shown to consist mainly of proanthocyanidins with epicatechin stereochemistry (^1^H NMR: B-ring protons: *ca*. 6.8 ppm, A-ring protons: *ca*. 6.0 ppm [21]; ^13^C NMR: C-2 at *ca*. 77 ppm, no signals at 80–82 ppm [22]). However, fraction Seph II (eluted with 40% MeOH) did not contain proanthocyanidins.

**Figure 4:**
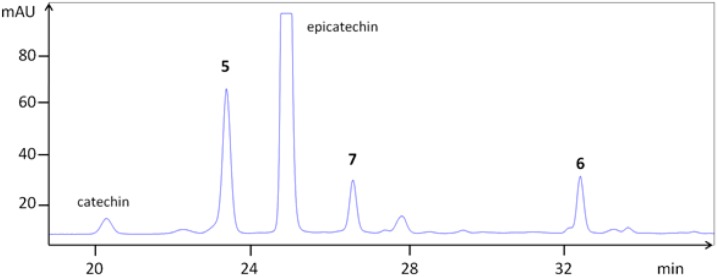
HPLC chromatogram of a bark extract of *Aronia melanocarpa* showing procyanidins B2 (**5**), B5 (**6**) and C1 (**7**).

In parallel to this procedure, dimeric (**5** and **6**) and trimeric (**7**) procyanidins (Figure 2) were isolated from Aronia bark. The bark was found to be a simpler source for isolation of procyanidins compared to berries, as the complexity with respect to total phenolic structures was lower (e.g., no anthocyanins). The concentration of procyanidins was higher in Aronia bark, as well. It has previously been shown that procyanidins B2, B5 and C1 are present both in bark and berries of the Aronia plant [2]. The isolated compounds were identified by co-chromatography against authentic substances (B2 and B5) and by mass spectrometry (Figure 4, Table 1). Thiolysis of crude extracts revealed that epicatechin was the major monomeric unit of the procyanidins, both as the starter and extender unit. Only minor amounts of catechin were detected. This might be due to epimerization of epicatechin.

**Table 1:** Chromatographic and spectral characterization of anthocyanins and procyanidins from *Aronia melanocarpa.*

	Compounds	TLC	HPLC	LCMS	[M + H]^+^	Fragments (am→u)
		rR_F_	*t*_R_ (min)	*t*_R_ (min)		
**1**	cyanidin 3-galactoside		12.9	22.0	449.104	287.044
**2**	cyanidin 3-glucoside		13.9	22.2	449.102	287.044
**3**	cyanidin 3-arabinoside		15.0	22.7	419.084	287.039
**4**	cyanidin 3-xyloside		17.8	24.3	419.085	287.040
	epicatechin	79	25.1	28.5	291.078	
**5**	epi-(4β→8)-epi (B2)	58	23.4	29.9	579.157	291.078
**6**	epi-(4β→6)-epi (B5)	67	32.5	35.6	579.168	291.084
**7**	epi-(4β→8)-epi-(4β→8)-epi (C1)	51	26.7	32.7	867.216	579.143, 291.080

It has previously been reported that polymeric procyanidins, composed predominantly of (−)-epicatechin units, are the major class of polyphenolic compounds in chokeberry [1,3]. The degree of polymerization of procyanidins varies from 2 to 23 units in the fruits, with clear domination of >decamers fraction and with flavan-3-ol subunits connected mainly with C4–C6 and C4–C8 bonds (B-type bonds) [1]. Free epicatechin is also present in black chokeberries, although its concentration is significantly lower in comparison with polymeric procyanidins. Previous investigations have reported anthocyanin concentrations of 0.6%–2% (DW) and procyanidin concentrations of 4%–5% (DW) in Aronia berries [2].

### 3.2. Biochemical Activities

The activity of crude extracts, subfractions and isolated compounds as DPPH scavengers, 15-LO and α-glucosidase inhibitors is shown in Table 2. The 50% EtOH crude extract showed high radical scavenging activity, and the effect was strengthened in the subfractions enriched in procyanidins (Amb-MeOH, Seph I and Seph III fraction). Trimeric procyanidin (compound **7**) showed higher radical scavenging activity than the dimeric procyanidins (compound **5** and **6**). The radical scavenging ability of compound **5** and **7** is in good accordance with the literature [23,24]. Compound **6**, however, seems to be previously unreported as a DPPH scavenger. Anthocyanins also possessed high radical scavenging capacity. IC_50_ values could not be established for compounds **1**–**4**, since an increase in sample concentration resulted in a strongly colored mixture that influenced the UV-absorbance. For this reason, percent scavenging at a sample concentration of 10.4 µg/mL was measured. Compound **1**–**3** were found to have the strongest DPPH scavenging capacity among the anthocyanins. The activity of compound **4** differed from the activity of the other anthocyanins, having the weakest DPPH radical scavenging capacity. Hence, sugar units linked to the anthocyanidin might have an influence on the biological effect. The radical scavenging activity of the anthocyanins is in fair accordance with previous investigations, although compound **3** has been reported to be slightly less active than **2** [25].

**Table 2:** Scavenging of the diphenylpicrylhydrazyl (DPPH) radical, 15-LO and α-glucosidase inhibitory activity of Aronia extracts, fractions and compounds.

Material	DPPH	α-Glucosidase	15-Lipoxygenase
	IC_50_ *^a^* (µg/mL) *^#^*/		
		IC_50 _ *^a^* (µg/mL)	IC_50_ *^a^* (µg/mL)
	% scavenging at 10.4 µg/mL *^^^*		
DCM	>167 *^#^*	Inactive	>83
EtOH	>167 *^#^*	Inactive	>83
50% EtOH	25.0 ± 5.0 *^#^*	3.5 ± 0.1	>83
Amb-MeOH	3.8 ± 0.2 *^#^*	0.55 ± 0.01	56.7 ± 0.7
Seph I	3.1 ± 0.5 *^#^*	nt *^b^*	30.3 ± 0.7
Seph II	12.0 ± 2.8 *^#^*	nt *^b^*	91.0 ± 4.8
Seph III	4.0 ± 0.5 *^#^*	nt *^b^*	33.0 ± 2.0
Compound **1**	39.0 ± 2.9 *^^^*	1.54 ± 0.1	71.5 ± 1.8
Compound **2**	37.0 ± 0.9 *^^^*	0.87 ± 0.2	73.3 ± 2.1
Compound **3**	40.0 ± 0.4 *^^^*	0.37 ± 0.08	58.7 ± 2.5
Compound **4**	25.0 ± 5.0 *^^^*	5.5 ± 1.6	>83
Compound **5**	4.7 ± 0.3 *^#^*	4.7 ± 0.2	65.1 ± 2.6
Compound **6**	5.2 ± 0.1 *^#^*	5.5 ± 0.1	72.3 ± 5.7
Compound **7**	3.2 ± 0.1 *^#^*	3.8 ± 0.2	57.6 ± 2.0
Quercetin (control)	3.0 ± 0.2 *^#^*	nt *^b^*	26.0 ± 2.0
Acarbose (control)	nt *^b^*	130.0 ± 20.0	nt *^b^*

*^a^* IC_50_: Concentration to give 50% scavenging or inhibition; *^b^* nt: Not tested; DCM: dichloromethane.

The 50% EtOH crude extract, the Amb-MeOH fraction and compound **1**–**7** showed high activity in the α-glucosidase assay compared to the positive control acarbose, an anti-diabetic drug. In addition, the purified anthocyanins were more active than the isolated dimeric and trimeric procyanidins (compound **5**–**7**). It is known that some anthocyanin extracts from plants exert a potent *in vitro* α-glucosidase inhibitory effect [26]. Also, McDougall *et al.* [27] found that the extent of inhibition of α-glucosidase is related to the anthocyanin content in different soft fruits. Among the anthocyanins, compounds **2** and **3** showed the highest activity and compound **4** the lowest. The activity of substance **1** is consistent with the literature [28]. To our knowledge, α-glucosidase inhibitory activity of substances **2**–**7** has not been reported previously. Ma *et al.* [29] showed that the α-glucosidase inhibitory activity of flavan-3-ol monomer and oligomers increased as the molecular weight increased, with a significant difference in potency between the strongest ones (pentamers) and the weakest one (monomer). The Amb-MeOH fraction appeared to contain polymeric procyanidins, and this could explain its strong effect towards α-glucosidase. Trimeric procyanidin (compound **7**) possessed stronger α-glucosidase inhibitory activity compared to the dimeric procyanidins (compound **5** and **6**). It appeared that the activity increased with increasing molecular weight, which is in good accordance with previously reported results [29]. For the anthocyanins, we found a highly significant correlation between α-glucosidase inhibition and DPPH radical scavenging activity (*p* < 0.005, *R*^2^ = 0.997). This is in good accordance with the literature [6]. For the crude extracts, the Amb-MeOH fraction and the procyanidins, the correlation was not significant. The Sephadex LH-20 fractions (Seph I–III) could not be tested for α-glucosidase inhibitory activity due to lack of material.

The Amb-MeOH fraction showed high inhibitory activity toward 15-LO, and the effect was strengthened in the subfractions enriched in procyanidins (Seph I and Seph III fraction). Differences in activity between isolated anthocyanins and procyanidins were relatively small. Both groups of compounds possessed high 15-LO inhibitory ability, with compound **3** and **7** being the most active ones. The 15-LO inhibition of **1**, **2**, **3** and **7** is in accordance with previous investigations [23,30]. Substances **4**, **5** and **6**, however, seem to be previously unreported as 15-LO inhibitors. 

The activity of crude extracts, subfractions and isolated compounds as XO inhibitors is presented in Table 3.

**Table 3:** Xanthine oxidase inhibitory activity of extracts, fractions and compounds from Aronia berries.

Material	% inhibition at a concentration of 42 µg/mL
DCM	Inactive
EtOH	Inactive
50% EtOH	Inactive
Amb-MeOH	26.3 ± 3.4
Seph I	32.2 ± 7.8
Seph II	19.9 ± 7.6
Seph III	46.5 ± 5.9
Compound **5**	12.7 ± 2.8
Compound **6**	6.6 ± 0.7
Compound **7**	15.5 ± 1.5
	**% inhibition at a concentration of 17 µg/mL**
Compound **1**	11.9 ± 4.4
Compound **2**	20.9 ± 3.4
Compound **3**	39.1 ± 2.1
Compound **4**	11.4 ± 2.8

The Amb-MeOH fraction possessed modest activity in the XO assay, and the effect was again strengthened in the subfractions enriched in procyanidins (Seph I and Seph III fraction). Due to absorbance above the upper detection limit of the spectrometer (sample concentrations >42 µg/mL for crude extracts, subfractions and procyanidins and sample concentrations >17 µg/mL for anthocyanins), higher concentrations of extracts and compounds could not be tested. Compound **7** was the strongest inhibitor among the isolated procyanidins, and compound **3** was the strongest among the anthocyanins. However, all were less efficient than the positive control quercetin (IC_50_ 0.6 ± 0.1 µg/mL). To our knowledge, inhibition of XO of Aronia berry extracts and substances **1**, **3**, **4** and **6** have not been reported previously. The 50% EtOH crude extract showed no inhibitory activity toward XO.

Both the DCM and the EtOH crude extract were inactive as DPPH radical scavengers, 15-LO, XO and α-glucosidase inhibitors. Among the isolated anthocyanins, compound **3** possessed the strongest and compound **4** the weakest radical scavenging and enzyme inhibitory activity. These effects seem to be influenced by the sugar units linked to the anthocyanidin. Trimeric procyanidin (compound **7**) showed higher activity in the biological assays compared to the dimeric procyanidins (compounds **5** and **6**), and it appeared that the activity increased with increasing molecular weight. In addition, there was a difference in activity between the two dimeric procyanidins, with compound **5** being the most active one. Reactive oxygen species can be generated by the prooxidative enzyme, XO, and the peroxidative enzyme, 15-LO, in vascular cells [31]. Components isolated from Aronia berries demonstrated inhibitory activity towards 15-LO and XO and may have a potential to alleviate oxidative stress. Until recently, anthocyanins were believed to have a very low bioavailability, but it has been demonstrated that the bioavailability of anthocyanins was underestimated [32,33]. In addition, anthocyanins are some of the few polyphenols that can be detected unmetabolized (e.g., as glycosides) in plasma [32]. It has to be taken into consideration that the bioavailability of flavanols varies depending on the degree of polymerization. Low molecular weight oligomeric procyanidins (DP ≤ 3) are absorbed intact in the gastrointestinal tract, but polymerization greatly impairs intestinal absorption [32,33,34]. In order to act as 15-LO and XO inhibitors *in vivo*, constituents have to be absorbed from the gastrointestinal tract. In view of the known bioavailability of tested compounds, Aronia products and extracts containing anthocyanins and oligomeric procyanidins (DP ≤ 3) may have biologically relevant 15-LO and XO effects. Inhibition of α-glucosidase delays carbohydrate digestion and the absorption of monosaccharides from the intestine [16]. Fractions enriched in procyanidins, the purified procyanidins and anthocyanins from *A. melanocarpa* berries were potent α-glucosidase inhibitors, suggesting that they may have beneficial effects in reducing blood glucose level. In order to act as α-glucosidase inhibitors *in vivo*, compounds do not have to be absorbed from the gastrointestinal tract, since it is a membrane-bound enzyme located at the epithelium of the small intestine [20]. Therefore, anthocyanins and procyanidins with even a high degree of polymerization may exert local effects in the gastrointestinal tract as α-glucosidase inhibitors.

## 4. Conclusions

We have shown that some extracts, fractions and constituents from *A. melanocarpa* possess activity of potential health benefits as radical scavengers, 15-LO inhibitors, XO inhibitors and α-glucosidase inhibitors *in vitro*. The difference in activity between the trimeric procyanidin C1 and the dimeric procyanidins B2 and B5 has, to our knowledge, not been reported previously. Also, it was found that the activity seems to be influenced by the sugar units linked to the anthocyanidin. No systematic investigations of *A. melanocarpa* extracts and fractions as radical scavengers, 15-LO inhibitors, XO inhibitors and α-glucosidase inhibitors has been reported previously. The activities of these extracts, fractions and compounds *in vivo* would seem to be an important subject for further research. The presence of biologically active compounds in Aronia berries increases the nutritional value of this plant, as a rich source of radical scavengers and inhibitors of peroxidative and prooxidative enzymes (15-LO, XO) and of α-glucosidase, an enzyme which may be involved in diabetes. This would appear to be of interest to the food industry.
